# Adenosine A1 and A2A Receptors in the Brain: Current Research and Their Role in Neurodegeneration

**DOI:** 10.3390/molecules22040676

**Published:** 2017-04-23

**Authors:** Jocelyn Stockwell, Elisabet Jakova, Francisco S. Cayabyab

**Affiliations:** Department of Surgery, University of Saskatchewan, Saskatoon, SK S7N 5E5, Canada; jocelyn.stockwell@usask.ca (J.S.); elisabet.j@usask.ca (E.J.)

**Keywords:** adenosine receptor cross-talk, A1R-mediated neurotoxicity, neurodegenerative diseases

## Abstract

The inhibitory adenosine A1 receptor (A1R) and excitatory A2A receptor (A2AR) are predominantly expressed in the brain. Whereas the A2AR has been implicated in normal aging and enhancing neurotoxicity in multiple neurodegenerative diseases, the inhibitory A1R has traditionally been ascribed to have a neuroprotective function in various brain insults. This review provides a summary of the emerging role of prolonged A1R signaling and its potential cross-talk with A2AR in the cellular basis for increased neurotoxicity in neurodegenerative disorders. This A1R signaling enhances A2AR-mediated neurodegeneration, and provides a platform for future development of neuroprotective agents in stroke, Parkinson’s disease and epilepsy.

## 1. Introduction

Adenosine signaling has been well studied in the brain and plays a complex role in multiple physiological and pathophysiological processes. Through a family of four G protein-coupled adenosine receptors, A1, A2A, A2B, and A3 [1], adenosine exerts neuromodulatory effects throughout the brain, affecting crucial processes such as normal neuronal signaling [2,3], astrocytic function [4,5,6], learning and memory [7,8,9,10], motor function [11], feeding [12], control of sleep [13], and normal aging processes [9,14,15]. Along with these normal physiological processes, adenosine is also involved in neuropathologies such as stroke [16], epilepsy [17], and Parkinson’s disease [18].

Of the four adenosine receptors, the A1 receptor (A1R) and A2A receptor (A2AR) are both highly expressed throughout the brain and have been widely studied [7,19]. This review explores current novel research into the function of these two receptors in the brain and their role in neurological diseases and neurodegeneration. Although the A1R has been traditionally described as a neuroprotective receptor due to its inhibitory effects [19], emerging evidence suggest that prolonged A1R activation may promote neurodegeneration [15,20,21]. This review explores the role of the A1R in the brain, including its normal physiological and pathophysiological effects and this emerging role as a neurodegenerative receptor, and how this may affect future studies. 

## 2. Adenosine Signaling in the Brain

Adenosine is an essential neuromodulatory molecule in the brain, but due to the widespread expression of adenosine receptors and the ubiquitous presence of adenosine, the complex role of adenosine signaling is not yet fully elucidated. A large body of research in recent years has given us a much better understanding of these receptors and their roles in the brain, although there is still much that is unknown. Adenosine exerts its action through activation of four G-protein coupled adenosine receptors, A1, A2A, A2B, and A3 receptors. Although the specific G proteins activated by each adenosine receptor are known, the intracellular effects of their activation are wide ranging and may vary based on cell function and location. In the brain, A1, A2B, and A3 receptors have widespread distribution, although A2B and A3 receptors have relatively low levels. However, A2ARs are primarily localized in the striatum, olfactory tubercle, and the nucleus accumbens [3]. In addition, these receptors have different affinities for adenosine, with the A1R having the highest affinity at approximately 70 nM and the A2AR having a lower affinity at approximately 150 nM [3]. The A2B and A3 receptors have a much lower affinity at 5100 nM and 6500 nM, respectively [3]. These affinities, along with differential expression of A1 and A2ARs in the brain, play a key role in these receptor actions in the brain. 

Adenosine receptors have been shown to have both a presynaptic and postsynaptic neuromodulatory effect. Presynaptically, adenosine modulates the release of neurotransmitters through both A1 and A2ARs [22]. A1Rs induce synaptic depression through reducing neurotransmitter release [23], whereas A2ARs are associated with increasing neurotransmitter release [24]. Postsynaptic activation of adenosine receptors causes decreased cellular excitability through activation of A1Rs, or increased excitability through A2ARs [25]. 

Recently, there has been much progress in expanding our understanding of the role of A1R activation in the brain. Through multiple second messenger pathways, adenosine exerts inhibitory effects throughout the brain. Activation of A1Rs has been shown to increase the activation of intracellular kinases and phosphatases including p38 mitogen-activated protein kinase (p38 MAPK) [20,26], C-jun N-terminal kinase (JNK) [20,27], and protein phosphatase 1, 2A and 2B (PP1, PP2A, PP2B) [21]. These intracellular actions affect transporters and receptors including AMPA receptors [15,20], NMDA receptors [28], ATP-sensitive K^+^ channels [29].

In the hippocampus, adenosine is predominantly inhibitory due to high expression levels of the high-affinity A1R [30]. Its role includes the modulation of learning and memory, including long-term potentiation (LTP) and long-term depression (LTD) [10,15]. A major mechanism by which adenosine modulates synaptic transmission is through modulation of excitatory glutamatergic neurotransmission [31]. Glutamate is a major excitatory neurotransmitter which induces synaptic transmission through activation of glutamate receptors, which are separated into two broad groups: metabotropic glutamate receptors (mGluRs) and ionotropic glutamate receptors. Ionotropic glutamate receptors include the ligand-gated AMPA, NMDA, and kainate receptors [32]. AMPA receptors (AMPARs) mediate fast excitatory synaptic transmission, whereas NMDA receptors are known to mediate slower excitatory synaptic transmission [33]. Recently, it has been shown that A1R activation induces the internalization of GluA1 and GluA2 subunit-containing AMPA receptors [15,20,21]. In other areas of the brain, including the substantia nigra (SN) and striatum, the lower affinity A2ARs are highly expressed, and have an excitatory neuromodulatory effect [34,35]. This excitatory effect may be in part due to the ability of the A2AR to increase the expression of the calcium-permeable GluA1 subunit of the AMPA receptor through the activation of PKA [36]. 

### 2.1. Regulation of Extracellular Adenosine Levels

The extracellular concentration of adenosine is tightly regulated, and changes in adenosine concentration can induce widespread effects in the brain. One mechanism through which adenosine is transported bidirectionally across cell plasma membranes is through equilibrative nucleoside transporters (ENTs), with ENT1 and ENT2 being the two best-described ENTs with ubiquitous tissue distribution compared to the remaining ENT3 and ENT4 subtypes [37]. Another class of nucleoside transporters expressed in mammalian cells is called concentrative nucleoside transporters (CNTs) which mediate the unidirectional co-transport of sodium and nucleosides against their concentration gradients [37,38]. 

During neuronal insult, such as in ischemia/hypoxia, the concentration of extracellular adenosine increases dramatically up to 100 times normal levels [39], with two possible sources: adenosine release from metabolically stressed ischemic cells into the extracellular space, and extracellular ATP metabolism [40]. As discussed above, this increased extracellular adenosine is dominantly inhibitory in the hippocampus [41]. It has been shown that A1Rs are highly activated in the hippocampus when extracellular adenosine levels increase [42,43]. ENTs are of particular interest due to reports implicating ENT modulation as a contributing factor in ischemia/hypoxia-induced neurodegeneration [44]. Regulation of extracellular adenosine in ischemic or hypoxic conditions by ENTs was suggested to be mediated by altering the surface expression of ENTs [45]. Additionally, Zhang, et al. (2011) later demonstrated that overexpression of the neuronal ENT1 reduced hypoxia/ischemia-induced increase in extracellular adenosine and suggested ENT1 as a potential therapeutic target for neuroprotection [46]. This reduction in extracellular adenosine is believed to be accomplished by uptake by ENT1 across cell membranes of neurons and other neighboring cells. This intracellular accumulation of adenosine is believed to be followed by either phosphorylation of adenosine back to adenosine monophosphate by adenosine kinase or its deamination to inosine by adenosine deaminase [47]. The predominant pathway for adenosine metabolism under physiological conditions appears to be adenosine phosphorylation by adenosine kinase, whereas under ischemic/hypoxic conditions when intracellular adenosine becomes markedly elevated, the deamination of adenosine becomes more predominant. Moreover, the released ATP during hypoxia or long bouts of excitation could be metabolized to adenosine by ecto-5′-nucleotidases. Hence, the levels of A1R and A2AR activation during hypoxic/ischemic events could be determined by any of these adenosine-related proteins. However, whether the so-called “purinomes” functionally interact with adenosine receptors and contribute to the cellular mechanisms of neurodegeneration, warrants further investigations.

### 2.2. Adenosine Receptors in Aging and Synaptic Plasticity

Adenosine has been shown to play a significant role in neuronal and cognitive changes that occur during natural aging processes. Aging is associated with cognitive deterioration [48], including memory loss and impaired induction of long-term potentiation (LTP), which has been reported in aging rats [49,50]. As the brain ages, the levels of extracellular adenosine increase [14,50]. However, the mechanisms by which these memory deficits develop during the aging process have not been fully examined. Recently we reported that enhanced adenosinergic signaling in aged brains leads to increased clathrin-mediated downregulation of AMPARs [15], which supports previous studies showing the importance of AMPARs in memory formation and impairments of LTP formation in aged rats [51,52]. In addition, activation of adenosine receptors modulates synaptic plasticity (e.g., LTP) differently in young, middle-aged and aged rats [9], and this differential modulation of LTP was attributed to a decreased efficiency of A1R-mediated regulation of synaptic transmission in aged rats [50]. Recent studies also reported that deficits in LTP induction in aged rats could also be mediated by altered levels of AMPARs and adenosine A1Rs and A2ARs [15]. This is consistent with previous results showing that high adenosine levels induced desensitization and downregulation of A1Rs in older brains [50,53]. We suggested that a novel mechanism that contributes to the regulation of the surface localization of adenosine receptors and AMPARs involves the physical interaction between A1Rs and AMPARs, which could lead to significant impairment in LTP in aged brains.

## 3. Role of A1Rs and A2ARs in Neurodegenerative Disease

Generally, A1Rs have been described as neuroprotective whereas A2ARs have been described as neurodegenerative [19]. This is largely due to the inhibitory effects of A1Rs and excitatory effects of A2ARs. Indeed, A2AR antagonism has shown promise in both preclinical and clinical research [54,55,56,57,58,59,60]. Additionally, non-selective adenosine receptor antagonists such as caffeine have also shown a neuroprotective role, which has been largely attributed to A2AR antagonism [7,48,61,62,63,64,65,66]. Table 1 outlines examples of preclinical adenosine receptor targeted drugs that have been used to study the effects of adenosine signaling in preclinical models of diseases such as ischemic stroke, epilepsy, and Parkinson’s disease. Indeed, due to increased understanding of the role of adenosine signaling in so many neuronal processes, adenosine-based therapies have been attractive for testing potential neuroprotection.

**Figure 1:**
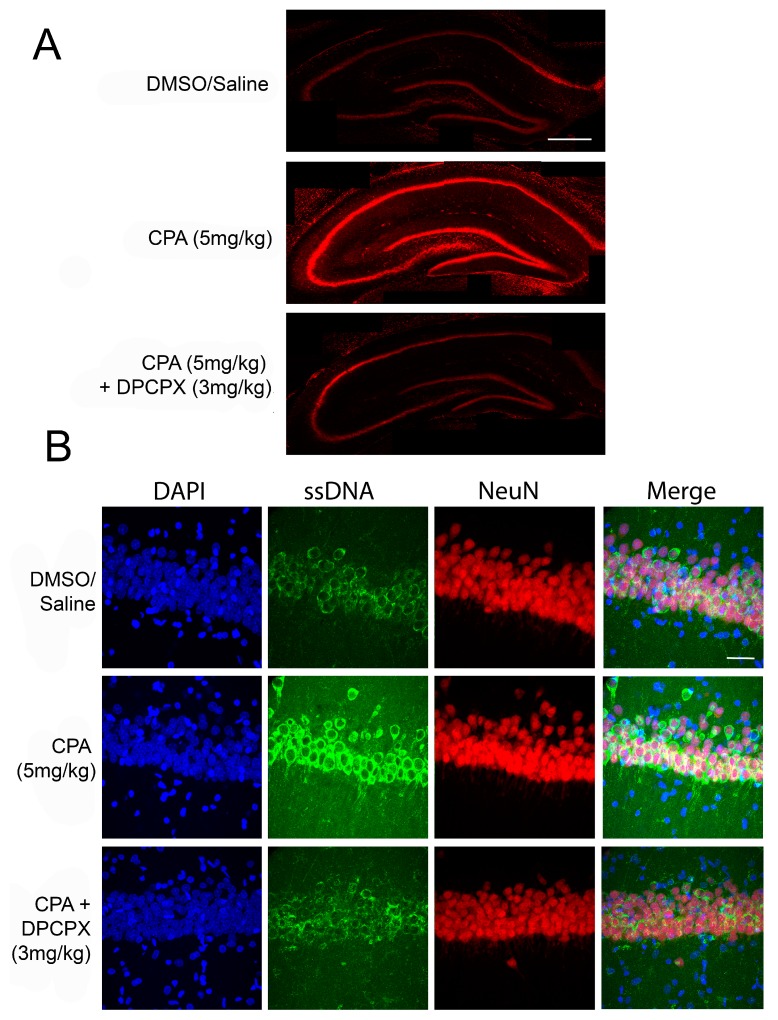
Adenosine A1R activation induces neuronal death in vivo. (**A**) Representative confocal microscopy images showing hippocampal slices stained with propidium iodide, a fluorescent marker for cell death. Male Sprague-Dawley rats were given intraperitoneal (i.p.) injections of CPA (5 mg/kg) or CPA (5 mg/kg) + DPCPX (3 mg/kg) and sacrificed at 48 h following initial injection. Acute coronal brain slices were taken and stained with propidium iodide. In animals treated with CPA alone, there was significantly increased propidium iodide fluorescence, indicating increased cell death in the hippocampus. DPCPX treatment prevented CPA-induced neuronal death. Scale bar 0.5 mm; (**B**) Confocal microscopy images of area CA1 of rat hippocampal slices with the same in vivo treatments above. DAPI, a nuclear stain is shown in blue (far left panels), single-stranded DNA (ssDNA) shown in green (second from left panels), NeuN shown in red (second from right panels), and a merge of all three channels shown in the far right panels. The marker ssDNA was used to label apoptotic cells, while NeuN (a neuronal marker) was used to label the CA1 cell layer. CPA treatment caused increased ssDNA staining in CA1 compared to control and DPCPX + CPA treated brains, indicating that CPA treatment was pro-apoptotic. Scale bar 30 µm.

In hypoxia or ischemia, extracellular adenosine increases up to 100-fold from extracellular ATP breakdown and adenosine extrusion from ischemic cells [39]. In fact, peripheral plasma adenosine has been shown to be elevated in humans up to 15 days after an ischemic stroke or transient ischemic attack [73], consistent with persistent elevation and action of adenosine in the brain. Induction of synaptic depression by A1R activation is thought to afford neuroprotection to ischemic cells by preventing excitotoxicity by reducing glutamate signaling caused by increased glutamate release that can occur in hypoxia/ischemia [74]. The role of adenosine in the induction of hippocampal synaptic depression in ischemia/hypoxia through adenosine A1 receptor (A1R) activation is thought to be neuroprotective by preventing excitotoxicity both presynaptically by inhibiting glutamate release [75] and postsynaptically by reducing cellular excitability [19]. However, recent studies suggest that A1R-mediated signaling pathways activated after stroke or ischemia could contribute to significant neuronal death (Figure 1) [20,21]. How A1R stimulation impacts on A2AR function in neurodegeneration is currently vigorously pursued by our group.

In epilepsy, the inhibitory A1R has been identified as an anti-epileptic receptor [17], whereas A2ARs have been shown to increase epileptiform activity [76]. Temporal Lobe Epilepsy (TLE) is a chronic form of epilepsy classified by the onset of partial seizures originating from the temporal lobe. Although the first line of treatment is anticonvulsant therapy, many patients are unresponsive to this and other established therapies [77,78], and over time, seizures induce damage to the brain and lead to cognitive and psychiatric problems [79]. A1R activation in TLE animal models has been shown to be protective against excitotoxicity and neuronal death in the hippocampus [80]. It has yet to be shown whether A1R activation is neuroprotective in human TLE patients. Recently, Fycompa (perampanel) is a new oral non-competitive AMPAR antagonist, which has been shown to be effective in treating drug-resistant partial onset seizures in patients ≥12 years old, and seizure control could be maintained for up to 2 years [81]. 

Prolonged A1R activation during hypoxia or focal cortical ischemia causes AMPAR endocytosis and persistent synaptic depression which could underlie hippocampal neurodegeneration [20,21]. However, subsequent normoxic reperfusion caused the lower-affinity A2ARs to increase the surface expression of GluA1 subunit-containing AMPARs [21], which are considered calcium-permeable AMPARs (CP-AMPARs). We propose that adenosine elevation in the brain and a prior A1R activation were required for the A2AR-mediated increase in GluA1 AMPARs [21], and that this signaling cross-talk could be involved in the seizure pathogenesis as well as hypoxia/ischemia-induced neurodegeneration. 

In Parkinson’s disease, A2ARs have been implicated in the pathology and development of the disease [82,83]. The loss of dopaminergic neurons in the substantia nigra and dopaminergic innervation of the striatum cause the well-known symptoms of Parkinson’s disease but the underlying cause of this neuronal loss is still largely unknown. The most commonly used therapeutics include Levodopa (L-Dopa, a dopamine precursor), dopamine receptor agonists, catechol-O-methyltransferase (COMT) antagonists, monoamine oxidase inhibitors, and antagonists of dopamine transporters [84,85]. These therapies slow the progression of disease symptoms in some patients, but unfortunately they are unable to prevent or reverse the progression of the disease. Although Levodopa is the gold standard therapy for Parkinson’s disease patients, chronic use of this drug leads to increased side effects including severe dyskinesia and increased “off time”, leading to the drug needing to be administered more often and at higher doses. Recently, clinical trials have explored the role of adenosine A2AR antagonists, namely Istradefylline, as an adjunct therapy to reduce these side effects, with recent success [57,83,86,87]. In addition, several on-going clinical trials are investigating the potential use of caffeine, a non-selective A1R and A2AR antagonist, in slowing the progression of PD symptoms [88,89]. 

It is believed that antagonizing A2ARs does not affect the dopaminergic system in healthy individuals, but regulates the GABAergic synaptic transmission in the basal ganglia. However, it is yet unknown how increased expression of A2ARs in the striatum or substantia nigra could contribute to Parkinson’s disease pathogenesis. Previous reports suggested that A2ARs and A1Rs can form heteromeric complex with D2 and D1 dopamine receptors [90,91,92], which suggests that increased adenosine levels in aging brain could lead to increased A2AR or A1R activation and subsequent downregulation of D1 and D2 receptor function. Moreover, it has also been suggested that increased A2AR function may be responsible for increased neuronal damage of dopaminergic neurons in the striatum [93], but the underlying mechanisms that lead to increased A2AR function and expression remain elusive.

A2ARs are known to interact with other receptors, such as D2 and D3 dopamine receptors and the metabotropic glutamate receptor mGluR5, forming functional heteromeric complexes [94,95]. Co-immunoprecipitation studies demonstrated the formation of A2A-D2, A2A-D3 and A2A-mGluR5 complexes [96,97]. Additionally, co-activation of the A2ARs and mGluR5 caused expression of the proto-oncogene c-Fos, phosphorylation of extracellular signal-regulated kinase (ERK), and dopamine- and cAMP-regulated neuronal phosphoprotein (DARPP-32), indicating a potential role of A2A-mGlu5 complexes in striatal plasticity. [98,99] The A2A-mGluR5 complex can also produce cellular effects on striatal neurons as demonstrated by a greater increase in GABA release from ventral striatopallidal neurons after perfusion of both A2A and mGluR5 agonists. [18,100]. In addition to these interactions, the recently suggested cross-talk between A1Rs and A2ARs [15,20,21] may also play a role in this aging-related disorder due to increased levels of adenosine in the brain, which may increase A2AR activation.

### 3.1. Clinical Testing of Adenosine Based Therapies 

In recent years, there have been a wide array of adenosine-based therapies tested in clinical trials for multiple diseases. Although there have been some successes, there have also been many drugs that have failed in clinical trials for various reasons. Table 2 outlines examples of recent clinically tested adenosine-based therapies, their mechanisms of action, and the success of the trial. To date, the only adenosine-based therapy being tested in clinical trials is the A2AR antagonist, Istradefylline, which is in Phase 3 clinical trials in Japan for Parkinson’s disease [57,86,87].

### 3.2. A1R Role in Neurodegeneration

Recently, there has been an emerging role of prolonged A1R activation as neurodegenerative in situations of drastically increased adenosine, such as in ischemic stroke. This lab has been studying the role of prolonged A1R activation both in vitro [15,20,21], and more recently, in vivo (unpublished). Following a pial vessel disruption (PVD) small vessel focal stroke model in rats [134,135], a decrease in surface A1Rs, an increase in A2ARs, and increased neuronal death was seen in the hippocampus 48h following stroke [20]. This was attributed to increased adenosine released in the brain, showing not only that adenosine can induce global damage in the brain following stroke, but that prolonged activation of the high-affinity A1R may lead to its internalization and the increase in A2AR expression, increasing neuronal excitability and potentially enhancing neuronal death. It was also found in a subsequent in vitro study that treatment with the A1R agonist CPA caused increased neuronal death in rat hippocampal slices [21], which was potentially due to decreased surface expression of the calcium-impermeable GluA2 subunit of the AMPA receptor and increased surface expression of the calcium-permeable GluA1 subunit of the AMPA receptor. Figure 1 shows exciting novel data suggesting that in vivo treatment with intraperitoneally injected CPA induces neurodegeneration in the rat hippocampus. Of note, these rats administered with CPA initially exhibited hypolocomotion and hypothermia, consistent with the widely reported central action of CPA or adenosine on A1R-mediated hypothermal response [3]; however, these rats readily recover within 1–2 h post i.p. CPA injection. Whereas endogenous adenosine may produce therapeutic hypothermia and hypometabolic state to induce neuroprotection in the short term (within minutes) after a stroke, we argue that prolonged actions (i.e., hours or days post stroke) of adenosine on A1Rs will promote neurotoxicity. Our novel finding (Figure 1) is the first evidence that prolonged A1R activation may lead to increased neuronal death in vivo. Currently, this lab is investigating this role of A1R-mediated neuronal death in ischemic stroke, epilepsy, and Parkinson’s disease, and the potential implications and mechanisms by which this adenosine-mediated neuronal death occurs. 

### 3.3. A1R/A2AR Cross-Talk

Cross talk between A1 and A2A receptors has been largely unexplored, but recent studies have suggested that there may be an intracellular interaction between these two receptors. Indeed, there are many instances of GPCR cross-talk [136,137]. Cross-talk between these two receptors was first explored by Lopes et al. when they suggested that A2ARs induce a PKC-mediated communication with A1Rs [138]. They showed that treatment of an A2AR agonist (CGS 21680), induced A1R desensitization, indicating that A2ARs had the potential to modulate A1R-mediated signaling. In areas of the brain where A2ARs are highly expressed, this may be a mechanism by which these two receptors exhibit cross-talk. 

**Figure 2:**
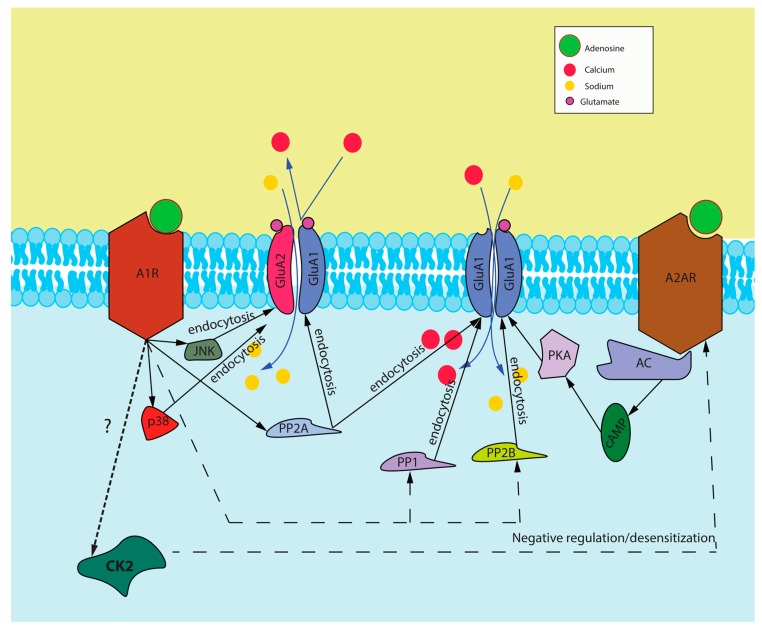
Proposed signaling cascade induced by A1R and A2AR activation. This figure represents our proposed interaction between A1Rs and A2ARs and how they interact to modulate the surface expression of AMPA receptors and also our proposed mechanism of cross-talk through CK2 activation. Abbreviations: A1R—adenosine A1 receptor, GluA1 and GluA2—subunits of AMPA receptors, A2AR—adenosine A2A receptor, JNK—C-jun N-terminal kinase, p38—p38 mitogen-activated protein kinase (MAPK), PP2A—protein phosphatase 2A, PP1—protein phosphatase 1, PP2B—protein phosphatase 2B, PKA—protein kinase A, cAMP—cyclic adenosine monophosphate, AC—adenylyl cyclase, CK2—protein kinase CK2.

In physiological conditions and in areas of the brain where the A1R is more highly expressed such as in the hippocampus and habenula, there is recent evidence that this cross-talk may occur in the opposite direction. There is evidence to suggest that increased A1R activation may increase the cell surface expression of A2ARs and may also induce A2AR-mediated upregulation of GluA1-containing AMPA receptors, thus increasing cellular calcium permeability and excitability [21]. However, we propose an alternative model (see Figure 2) whereby A1R and A2AR signaling could be linked by the serine/threonine protein kinase CK2 (formerly casein kinase 2). This protein kinase has been observed to be downregulated in several neurodegenerative disorders, including Alzheimer’s disease, ischemia, and Parkinson’s disease [139]. Pilot studies (Chen and Cayabyab, unpublished) suggest that this CK2 is downregulated in our focal cortical stroke model evoked by pial vessel disruption. Since we previously reported [20] that A1Rs are downregulated while A2ARs are upregulated after focal cortical ischemia, and previous reports by others demonstrating that CK2 negatively regulates A2AR desensitization rate [140], it is therefore plausible to suggest that the observed downregulation of CK2 in our focal cortical ischemic stroke model could be mediated in part by the prolonged A1R activation and subsequent A1R desensitization after stroke. We propose that this is followed by downregulation of CK2, which in turn leads to decreased desensitization of A2ARs. The consequent increase in A2AR surface expression could play a major role in neurodegeneration in vulnerable brain regions in Parkinson’s disease, epilepsy and stroke.

## 4. Summary and Conclusions

The actions of adenosine in the brain are widespread and complicated, and are still not fully elucidated. In recent years, understanding of the role of the adenosine A1R and A2AR in both normal physiological conditions and in neurodegenerative diseases has grown substantially, and novel research may allow for the identification of better therapeutic strategies. This review outlines some novel research in the role of adenosine A1 and A2A receptors in the brain in both normal and pathological conditions, and outlines recent advances in the understanding of the role of adenosine receptors in the brain. Recently, there has been an emerging neurodegenerative role for prolonged A1R activation that may lead to a reevaluation of current adenosine based strategies in multiple neurodegenerative diseases, and may also allow for a better understanding of how this receptor regulates neuronal function.

## 5. Future Perspectives 

A major focus of our lab is to elucidate the mechanism by which the apparent A1R/A2AR cross-talk occurs, and how this affects neurodegeneration in multiple disorders, including ischemic stroke, epilepsy, and Parkinson’s disease. Additionally, we are exploring the intracellular effects of prolonged A1R activation and its role in neuronal death. In Parkinson’s disease, for example, preliminary data suggest that mitochondrial dysfunction may be induced by prolonged A1R activation and that this may lead to increased tyrosine hydroxylase dysregulation. This mitochondrial dysfunction may also lead to lipid dyshomeostasis through altering lipid translocation or gene regulation. In epilepsy, we are exploring the role of A2AR-mediated neurodegeneration and the potential role of A1Rs in modulating both the induction and damage caused by epileptiform activity. We propose that use of a genetic A1R knockout may help further explore the role of A1Rs in the regulation of AMPA receptor regulation, A2AR signaling and cross-talk, and in the development of adenosine-mediated neuronal damage. 

## Figures and Tables

**Table 1:** Name, mechanism of action (MoA), use in preclinical trials and potential role of Adenosine A1, A2A, and A3 receptor agonists and antagonists.

Drug Name	MoA	Preclinical Trial	Results
**2-HE-NECA**	A2AR agonist	Epilepsy prone transgenic mouse strain (DBA/2 strain) [67]	Suppresses seizure activity of both tonic and clonic extension seizures
**CCPA**	A1R agonist	Epilepsy prone rats (GEPR-9 strain) [68]	2. Suppresses seizure activity
**CGS 21680**	A2AR agonist	1. Bicuculline methiodide-induced motor seizures in male Sprague-Dawley rats [69]	1. Inefficient antagonist of bicuculline-induced seizures
		2.Epilepsy prone transgenic mice (DBA/2 strain) [67]	2. Suppresses seizure activity, of both tonic and clonic extension seizures
**Cl-IB-MECA**	A3R agonist	Epilepsy prone mice (DBA/2 strain) [67]	Ineffective anti-epileptic
**CPA**	A1R agonist	Pentylenetetrazole-induced seizures in Wistar rats [70]	Significant protection against pentylenetetrazole-induced seizures
**CPCA**	A2AR agonist	1. Pentylenetetrazole-induced seizures in Wistar rats [70]	1. Ineffective anti-epileptic
		2. Epilepsy prone rats (GEPR-9 strain) [70]	2. Suppresses seizure activity
**DMPX**	A2AR antagonist	Pentylenetetrazole-induced seizures in Wistar rats [71]	Kept protection afforded by CPA against Pentylenetetrazole-induced seizures
**DPCPX**	A1R antagonist	Pentylenetetrazole-induced seizures in Wistar rats [70]	Reverse protection afforded by CPA against Pentylenetetrazole-induced seizures
**MRS1523**	A3R antagonist	Ex vivo seizure activity in hippocampal slices from Sprague-Dawley rats [72]	Reduced both seizure duration and intensity
**Theophylline**	Nonspecific adenosine receptor antagonist	Pentylenetetrazole-induced seizures in Wistar rats [70]	Reverse protection afforded by CPA against Pentylenetetrazole-induced seizures
**ZM 241385**	A2AR antagonist	Ex vivo seizure activity in hippocampal slices from Sprague-Dawley rats [72]	Shorten the duration of epileptiform activity

*Abbreviations:* 2-(1-Hexyn-1-yl) adenosine-5′-*N*-ethyluronamide (**2-HE-NECA**); 8-(p-Sulfophenyl) theophylline hydrate (**8-SPT**); 2-Chloro-*N*^6^-cyclopentyladenosine (**CCPA**); 3-[4-[2-[[6-amino-9-[(2*R*,3*R*,4*S*,5*S*)-5-(ethylcarbamoyl)-3,4-dihydroxy-oxolan-2-yl]purin-2-yl]amino]ethyl] phenyl] propanoic acid (**CGS 21680**); 2-chloro-*N*(6)-(3-iodobenzyl) adenosine-5′-*N*-methylcarboxamide (**Cl-IB-MECA**); *N*(6)-Cyclopentyladenosine (**CPA**); 3α-carbomethoxy-4β-(4-chlorophenyl)-*N*-methylpiparidine (**CPCA**); 3,7-dimethyl-1-propargylzanthine (**DMPX**); 8-cyclopentyl-1,3-propargylzanthine (**DPCPX**); 3-Propyl-6-ethyl-5-[(ethylthio)carbonyl]-2 phenyl-4-propyl-3-pyridine carboxylate (**MRS1523**); and 4-(2-(7-amino-2-(furan-2-yl)-[1,2,4]triazolo[1,5-α][1,3,5]triazin-5-ylamino)ethyl)phenol (**ZM 241385**).

**Table 2:** Name, mechanism of action (MOA), use in Clinical trials and potential role of Adenosine A1, A2A, and A3 receptor agonists and antagonists.

Drug Name	MOA	Clinical Trial	Results
**Adenosine**	Non selective agonist	1. The role of adenosine in the release of VEGF and Cytokines, Phase 1 [101]	1. (NCT00580905) * Terminated
		2. A possible therapeutic role for adenosine during inflammation, Phase 1 [102,103]	2. (NCT00513110) Completed
		3. Prophylactic intra-coronary adenosine to prevent post coronary artery stenting myonecrosis, Phase 3 [101]	3. (NCT00612521) Terminated
		4. Postconditioning with adenosine for ST-elevated myocardial infarction, Phase 2 [104]	4. (NCT00284323) Ongoing
		5. Myocardial protection with adenosine during primary percutaneous coronary intervention in patients with ST-elevated myocardial infarction, Phase 3 [105]	5. (NCT00781404) Completed
		6. Clonidine versus adenosine to treat neuropathic pain, Phase 2 [106]	6. (NCT00349921) Completed
		7. Dose response of adenosine for perioperative pain, Phase 2 [107]	7. (NCT00298636) Completed
		8. Perioperative ischemia-induced liver injury and protection strategies [108]	8. (NCT00760708) Ongoing
**Apadenoson**	A2AR agonist	Adenosine 2A agonist lexiscan in children and adults with sickle cell disease, Phase 1 [109]	(NCT01085201) Completed
**Caffeine**	Non selective antagonist	1. Caffeine for motor manifestations of Parkinson’s disease, Phase 2	1. (NCT01190735) Completed
		2. Study investigating caffeine for excessive daytime somnolence if Parkinson’s disease, Phase 2 & 3	2. (NCT00459420) Completed
		3. Caffeine as a therapy for Parkinson’s disease, Phase 3 [88]	3. (NCT01738178) Ongoing
**CF-101**	A3R agonist	1. Safety and efficacy study of CF101 to treat Psoriasis, Phase 2 [110]	1. (NCT00428974) Completed
		2. Oral CF101 tablet and methotrexate treatment in Rheumatoid arthritis patients, Phase 2 [111]	2. (NCT00556894) Completed
**CF-102**	A3R agonist	A phase 1-2 Study of CF102 in patients with advanced hepatocellular carcinoma, Phase 1 & 2 [112]	(NCT00790218) Completed
**Dipyridamole**	Adenosine uptake inhibitor	1. “Normal coronary artery” with slow flow improved by adenosine injection, dipyridamole treatment, and clinical follow-up, Phase 1	1. (NCT00960817) Recruitment status unknown
		2. Clinical trial of dipyridamole in Schizophrenia [90,113,114]	2. (NCT00349973) Completed
		3. Can dipyridamole induce protection against ischemia and reperfusion injury in patients undergoing elective coronary artery bypass grafting, Phase 4 [115]	3. (NCT01295567) Competed
		4. Circulating adenosine levels before and after Intravenous (IV) persantine [101]	4. (NCT00760708) Terminated
		5. A phase II trial comparing Z-102 with placebo in patients with moderate to severe rheumatoid arthritis, Phase 2 [101]	5. (NCT01369745) Completed
**GW493838**	A1R agonist	The study of GW493838, an adenosine A1 receptor agonist, in peripheral neuropathic pain, Phase 2 [101]	(NCT00376454) Completed
**INO 8875**	A1R agonist	1. A dose-escalation study designed to evaluate the tolerability, safety, pharmacokinetics, and efficacy of chronic topical ocular application of INO-8875 in adults with ocular hypertension or primary open-angle glaucoma, Phase 1 [101]	1. (NCT01123785) Completed
		2. Study of trabodenoson in adults with ocular hypertension or primary open-angle glaucoma, Phase 3	2. (NCT02565173) Completed
**Istradefylline**	A2AR antagonist	1. Study of Istradefylline for the treatments of Parkinson’s disease in patients taking levodopa, Phase 3 [83,116]	1. (NCT00955526) Completed
		2. Long-term study of Istradefylline in Parkinson’s disease patients, Phase 3 [87]	2. (NCT00957203) Completed
		3. A 12-week randomized study to evaluate oral Istradefylline in subjects with moderate to severe Parkinson’s disease, Phase 3	3. (NCT01968031) Completed
		4. The effects of mild Hepatic impairment on the pharmacokinetics of Istradefylline, Phase 1 [86]	4. (NCT02256033) Completed
		5. An extension of Istradefylline in North American Parkinson’s disease patients who have completed study 6002-INT-001, Phase 3	5. (NCT00199381) Terminated
		6. The effects of rifampin on the metabolism of Istradefylline in healthy volunteers, Phase 1	6. (NCT02174250) Completed
**Preladenant**	A2AR antagonist	1. A placebo- and active-controlled study of preladenant in early Parkinson’s disease, Phase 3	1. (NCT01155479) Terminated
		2a. A placebo- and active-controlled study of preladenant in subjects with moderate or severe Parkinson’s disease, Phase 3 [117]	2.a (NCT01155466) Completed
		2b. An active-controlled extension study to NCT01155466 [P04938] and NCT01227265 [P07037], Phase 3	2.b (NCT01215227) Terminated
		3. A placebo controlled study of preladenant in participants with moderate to severe Parkinson’s disease, Phase 3 [117]	3. (NCT01227265) Completed
		4. A dose finding study of preladenant for the treatment of Parkinson’s disease, Phase 2 [118]	4. (NCT01294800) Completed
**Regadenoson**	A2AR agonist	1. Advance MPI2: Study of regadenoson versus adenoscan in patients undergoing myocardial perfusion imaging, Phase 3 [119]	1. (NCT00208312) Completed
		2. Myocardial perfusion magnetic resonance imaging using regadenoson, Phase 1 [119,120,121]	2. (NTC00881218) Completed
		3a. Adenosine 2A agonist lexiscan in children and adults with sickle cell disease, Phase 1 [109]	3.a (NCT01085201) Completed
		3b. A phase II trial of regadenoson in sickle cell anemia, Phase 2 [122]	3.b (NCT01085201) Currently recruiting
		4. Microvascular blood flow in sickle cell anemia [123,124]	4. (NCT01566890) Currently recruiting
		5. Regadenoson blood flow in type 1 diabetes, Phase 4 [125]	5. (NCT01019486) Completed
**Rolofylline**	A1R antagonist	1. Protect-1, A study of the selective A1 adenosine receptor antagonist KW-3902 for patients hospitalized with acute HF and volume overload to assess treatment effect on congestion and renal function, Phase 3 [126,127]	1. (NCT00328692) Completed
		2. Protect-2, A study of the selective A1 adenosine receptor antagonist KW-3902 for patients hospitalized with acute HF and volume overload to assess treatment effect on congestion and renal function, Phase 3 [127,128,129]	2. (NCT00354458) Completed
**SYN-115**	A2AR antagonist	1. An fMRI study of SYN-115 in cocaine dependent subjects [130,131]	1. (NCT00783276) Completed
		2. Safety and efficacy study of SYN-115 in Parkinson’s disease patients using levodopa to treat end of dose wearing off, Phase 2 & 3 [132]	2. (NCT01283594) Completed
**Tonapofylline**	A1R antagonist	Study to assess the safety and tolerability of IV tonapofylline in subjects with acute decompensated heart failure and renal insufficiency, Phase 2 [133]	(NCT00709865) Completed

* Indicates Clinical trials identifier (ClinicalTrials.gov); *Abbreviations: N*(6)-(3-iodobenzyl) adenosine-5′-*N*-methylcarboxamide or IB-MECA (**CF-101**); 2-chloto-*N*(6)-(3-iodobenzyl) adenosine-5′-*N*-methylcarboxamide or Cl-IB-MECA (**CF-102**); 2S,3S,4R,5R)-2-(5-tert-Butyl-1,3,4-oxadiazol-2-yl)-5-(6-(4-chloro-2-fluoro-anilino)purin-9-yl)tetrahydrofuran-3,4-diol (GW493838); Trabodenoson (**INO 8875**); and Tozadenant (**SYN-115**).
